# Magnetic resonance of the heart in a muscular dystrophy patient with an MR conditional ICD: Assessment of safety, diagnostic value and technical limitations

**DOI:** 10.1186/1532-429X-15-49

**Published:** 2013-06-11

**Authors:** Anca Florian, Anna Ludwig, Sabine Rösch, Udo Sechtem, Ali Yilmaz

**Affiliations:** 1 From the Department of Cardiology and Angiology, University Hospital Münster, Albert-Schweitzer-Campus 1, Gebäude A1, 48149 Münster, Germany; 2 From the Division of Cardiology, Robert-Bosch-Krankenhaus, Stuttgart, Germany

**Keywords:** MR-conditional ICD, Cardiovascular magnetic resonance, Cardiomyopathy, Muscular dystrophy

## Abstract

Cardiovascular magnetic resonance (CMR) studies in patients with pacemakers or implantable cardioverter/defibrillators (ICD) are increasingly required in daily clinical practice. Therefore, in the last years the manufacturers developed not only MR-conditional pacemakers, but also MR-conditional ICDs. However, the clinical experience regarding the feasibility and limitations of MR studies of the heart in patients with ICDs is still limited. In particular, there are hardly any CMR studies in the same patients performed prior to and post ICD implantation allowing a one-to-one comparison of the obtained CMR images. This is the first presentation of a CMR study in a patient with the world’s first and so far only MR-conditional ICD. In our case, a major problem related to the presence of the MR conditional ICD was an image artifact caused by the device’s generator which hampered the visualization of the midventricular and apical anterior and antero-lateral segments in all sequences performed. Considering previous studies, right chest implantation of the ICD could probably have helped in this setting and may be preferred in future ICD implantations. Our case report nicely illustrates the real clinical need for specially designed implantable devices that ensure safe and high-quality imaging in patients in whom serial CMR is required.

## Background

Becker muscular dystrophy is an inherited, recessive, X-linked dystrophin deficiency disease with similar, but more benign clinical picture compared to Duchenne muscular dystrophy. Cardiac involvement with dilated cardiomyopathy and ventricular arrhythmias are frequent findings. In muscular dystrophy patients with cardiac involvement, imaging by cardiovascular magnetic resonance (CMR) - in addition to current standard of care (monitoring by ECG and echocardiography) - brings several advantages: By providing accurate and reproducible measurements for left ventricular (LV) volumes and function as well as by visualizing the characteristic non-ischemic pattern of late gadolinium enhancement (LGE) in the infero-lateral wall, CMR represents a sensitive tool for early diagnosis, monitoring of disease progression and response to treatment [1]. Conventionally, the presence of a pacemaker or an implantable cardioverter/defibrillator (ICD) was considered an absolute contraindication for CMR. Several studies have addressed the issue of performing CMR in patients with non-MR-conditional cardiac rhythm devices. They found CMR to be safe in appropriately selected patients with close monitoring and special precautions related to device programming [2-4]. Besides safety issues, image quality and analysis can be variably impaired primarily by the presence of susceptibility artifacts associated with the generator on the chest. Recently, MR conditional pacemakers and ICDs have been designed to overcome the existing limitations [5].

## Case presentation

A 26-year-old male patient with Becker muscular dystrophy and secondary dilated cardiomyopathy received an MR-conditional ICD (ICD; Biotronik Lumax 740 VR-T DX) for primary prevention of sudden cardiac death. Screening by echocardiography and CMR had revealed cardiac involvement already three years ago and etiology was confirmed by endomyocardial biopsy with immunohistochemical proof of myocardial dystrophin deficiency at that time. The patient was still ambulatory, presenting moderate muscle weakness with difficulties in climbing stairs and mild to moderate exertional dyspnea. Before ICD implantation, he underwent regular follow-up echocardiography and CMR studies (every year) as a participant in an ongoing research project on patients with muscular dystrophy.

### CMR prior to ICD implantation

**Figure 1:**
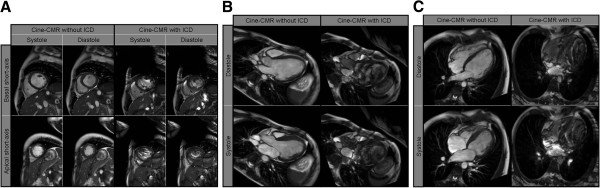
**Comparative cine-CMR images prior to and post ICD-implantation.** Comparative cine-CMR (SSFP) still images: at end-diastole, respective end-systole, in basal and apical cardiac short axis (**A**), 3-chamber (**B**) and 4-chamber views (**C**) before (left panels) and after ICD implantation (right panels). A large susceptibility artifact coming from the ICD generator, situated on the left anterior hemithorax, blurs the visualization of the anterior and antero-lateral left ventricular myocardial segments (right panel).

**Figure 2:**
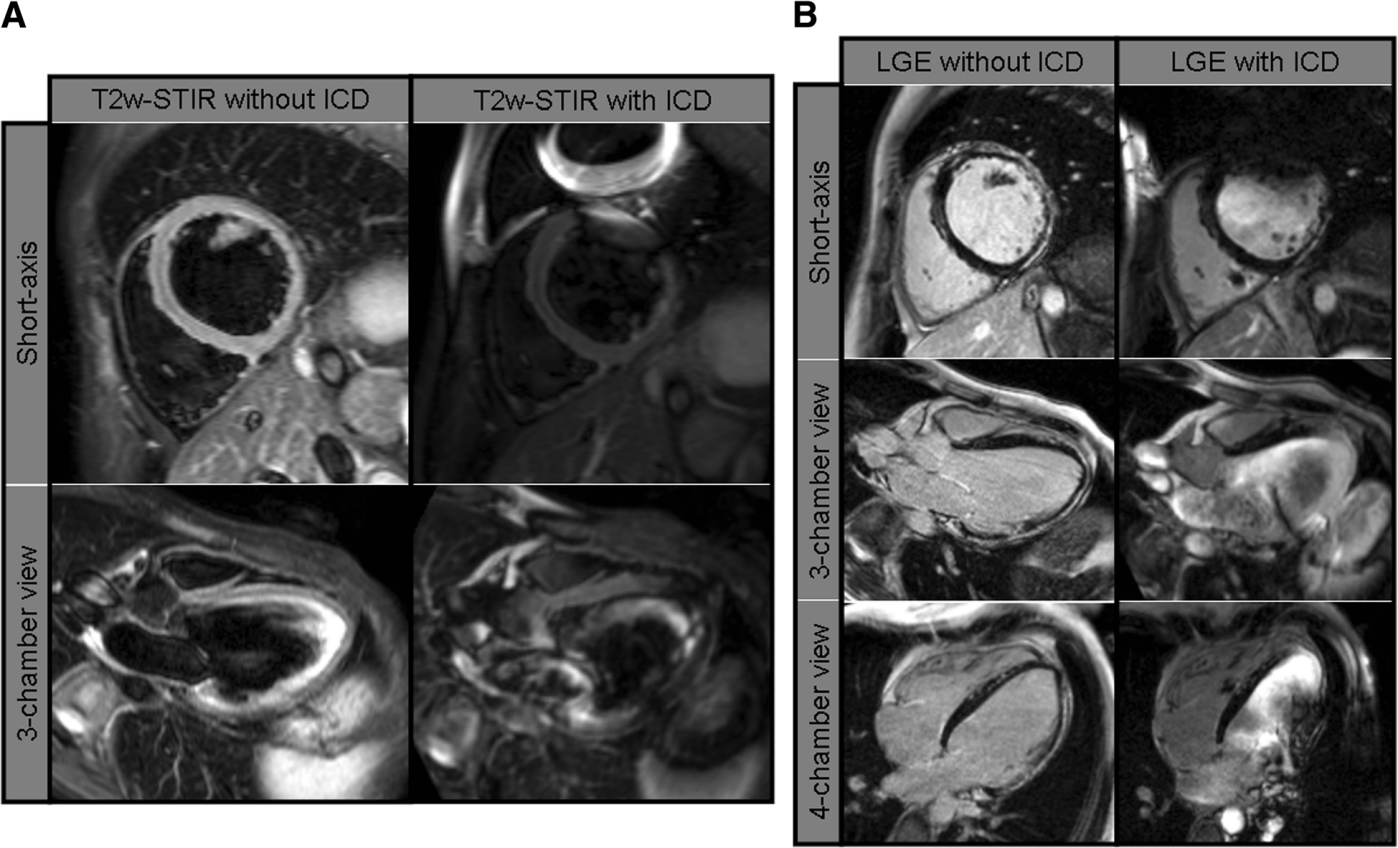
**Edema and contrast images prior to and post ICD-implantation. A**. T2-weighted Edema and contrast images prior to and post ICD-implantation. T2-weighted “edema” images (T2w-STIR spin-echo) in midventricular short-axis and 3-chamber view before (left panel) and after ICD implantation (right panel). Similarly to the cine-CMR, artifacts from the ICD render the anterior and antero-lateral segments not assessable (right panel). **B**. Late gadolinium enhancement (LGE) images prior to and post ICD-implantation. Late gadolinium enhancement (LGE) images in mid-ventricular short-axis, 3-chamber and 4-chamber views before (left panel) and after ICD implantation (right panel).

Prior to ICD implantation, resting electrocardiogram (ECG) showed sinus rhythm, normal duration QRS with left anterior fascicular block and deep lateral Q waves. Transthoracic echocardiography showed a dilated LV with severely impaired systolic function (LV ejection fraction (LV-EF) = 35% by Simpson’s method) due to hypo-/akinesia predominantly in the left ventricular free wall. Repeated ambulatory ECG monitoring showed frequent non-sustained ventricular tachycardia episodes. For further characterization of LV function and morphology, additional CMR studies were performed on a 1.5-T scanner (Siemens Aera, Erlangen, Germany). In the last study prior to ICD implantation, functional analysis by SSFP-cine sequences showed a dilated LV (LV end diastolic volume (LVEDV) = 146 mL/m^2^, LV end systolic volume (LVESV) = 95 mL/m^2^) with severely impaired systolic function (LV-EF = 35%) due to diffuse hypokinesia, highly pronounced in the inferior and lateral free wall (Figure 1A-C, left panels; Movies 1A-C in Additional file 1). In addition, a non-dilated right ventricle with normal systolic function was found. T2-weighted edema imaging (T2w-STIR spin-echo) was negative for myocardial edema (Figure 2A, left panels). LGE-imaging showed extensive subepicardial enhancement in the inferior and lateral walls and mid-myocardial enhancement in the apical half of the septum (Figure 2A, left panels). Due to a) the important added value of the CMR-derived information regarding the pattern and severity of myocardial disease and b) the necessity for close follow-up, a “MR conditional” single chamber ICD was implanted in 2011.

### CMR post ICD implantation

This year, the patient presented again for cardiac follow-up and underwent his first follow-up CMR study six months after ICD implantation (as part of the study protocol). There were no changes in physical examination, ECG and echocardiography findings. No sustained arrhythmic events, nor ICD therapy were recorded since implantation. Before performing CMR, device interrogation was performed and both ICD as well as pacemaker functions were switched off. Scanning was performed on the same scanner as the initial study with the same imaging protocol. Only the specific absorption rate (SAR) was restricted to 2.0 W/kg. The patient was closely monitored (ECG and pulse-oximetry) during the whole stay in the MR environment. During the scan, the patient was completely asymptomatic and no arrhythmic events were recorded. Image quality was limited compared to the previous study without an ICD due to substantial susceptibility/banding artefacts associated with the ICD generator on the left hemithorax (Figures 1 and 2). Notably, there was no artefact caused by the right ventricular electrode. Cardiac function (Figure 1A-C, right panel), myocardial edema (Figure 2A, right panel Movies 1A-C in Additional file 1) and presence of LGE (Figure 2B, right panel) could be assessed but it was impossible to visualize the midventricular and apical anterior and antero-lateral segments in a meaningful way. However, the remaining segments could be evaluated without limitations and demonstrated no changes compared to the previous CMR examination. After imaging, the device was interrogated and reprogrammed with the initial settings. Lead impedance and function were unaffected after the scan. High-sensitive troponin I levels demonstrated normal values prior to and after the examination ruling out a myocardial damage due to overheating of the electrode tip.

## Conclusion

To the best of our knowledge, this is the first presentation of MR of the heart in a patient with the world’s first and so far only MR-conditional ICD. In our case, a major problem related to the presence of the MR conditional ICD was an image artifact caused by the device’s generator which hampered the visualization of the midventricular and apical anterior and antero-lateral segments in all sequences performed. Considering previous studies, right chest implantation of the ICD could probably have helped in this setting and may be preferred in future ICD implantations [6]. In the meantime, Biotronik has gained CE approval for two more MR-conditional ICD models (Ilesto7 and Iforia). Since the generator of the model Iforia is relatively small, it may help to reduce the aforementioned artefacts. Our case report nicely illustrates the real clinical need for specially designed implantable devices that ensure safe and high-quality imaging in patients in whom serial CMR is required.

## Consent

Written informed consent was obtained from the patient for publication of this case report and any accompanying images. A copy of the written consent is available for review by the Editor-in-Chief of this journal.

## Abbreviations

CMR: Cardiovascular magnetic resonance; ICD: Implantable cardioverter/defibrillator; LGE: Late gadolinium enhancement; LV: Left ventricle; LVEDV: Left ventricular end diastolic volume; LVESV: Left ventricular end systolic volume; SAR: Specific absorption rate.

## Competing interests

The authors declare that they have no competing interests.

## Authors’ contributions

AF planned the scan protocol, assisted in image acquisition and interpretation, and helped to draft the manuscript. AY provided cardiology supervision during scanning, reviewed device interrogations, assisted in image acquisition and interpretation, and drafted the manuscript. AL and SR assisted with preparing the patient and planning as well as performing the scan protocol. US provided additional supervision and critically reviewed the manuscript. All authors read and approved the final manuscript.

## Supplementary Material

Additional file 1 Movies 1A-C. Click here for file
